# Detection of myocardial scar using T2* in a clinical setting

**DOI:** 10.1186/1532-429X-17-S1-Q12

**Published:** 2015-02-03

**Authors:** Britta Butzbach, Bernhard Schnackenburg, christoph jacoby, Malte Kelm, Mirja Neizel-Wittke

**Affiliations:** 1 Department of Cardiology, Duesseldorf, Germany; 2 Philips Healthcare, Hamburg, Germany

## Background

Detection of late gadolinium enhancement (LGE) is the method of choice for imaging myocardial scar. However, patients who suffer from cardiovascular disease often are afflicted with renal impairment. As a GFR <30 ml/min bears the risk of nephrogenic systemic fibrosis it would be beneficial to develop alternative cardiac imaging methods without contrast agent. De Jong et al have already used UTE (ultra short echo time) to detect myocardial scar with a fast T2* signal decay in a rat infarct model. The aim of the study was to validate UTE imaging to detect myocardial scar without contrast agent in patients with coronary artery disease.

## Methods

45 patients (75 +-10 years) with coronary artery disease and known myocardial scar were enrolled in the study. Cardiac MRI was conducted on a 1.5T Philips Archieva scanner with a standard cine balanced steady state free precession sequence for acquiring left and right ventricular volumes. Also, UTE was conducted with long (6 ms) and short TE (0.6 ms) for the imaging of fibrosis without contrast agent. LGE images were acquired for comparison of scar imaging with UTE-images. Measurements were compared with LGE images regarding fibrotic or scar tissue-area and signal intensity. Images were analyzed using Osirix 5.9 software.

## Results

27 Patients of 45 were analyzed. 18 date sets were excluded due to poor image quality. There was a good correlation regarding fibrotic and scar tissue-area in UTE compared to conventional late gadolinium enhancement (r= 0.9, p<0,0001). There was good signal intensity of UTE-acquired images (t-test p<0.0001).

## Conclusions

In-vivo imaging of myocardial scar is feasible without contrast agent in patients with coronary artery disease. This is a great advantage for patients with renal impairment and contraindication for gadolinium contrast agent. In current status, UTE-images are only feasible with one slice imaging. Further work on the sequence need to be done, to image the whole left ventricle with UTE for scar imaging.

## Funding

Funded by the University of Duesseldorf.

**Figure 1:**
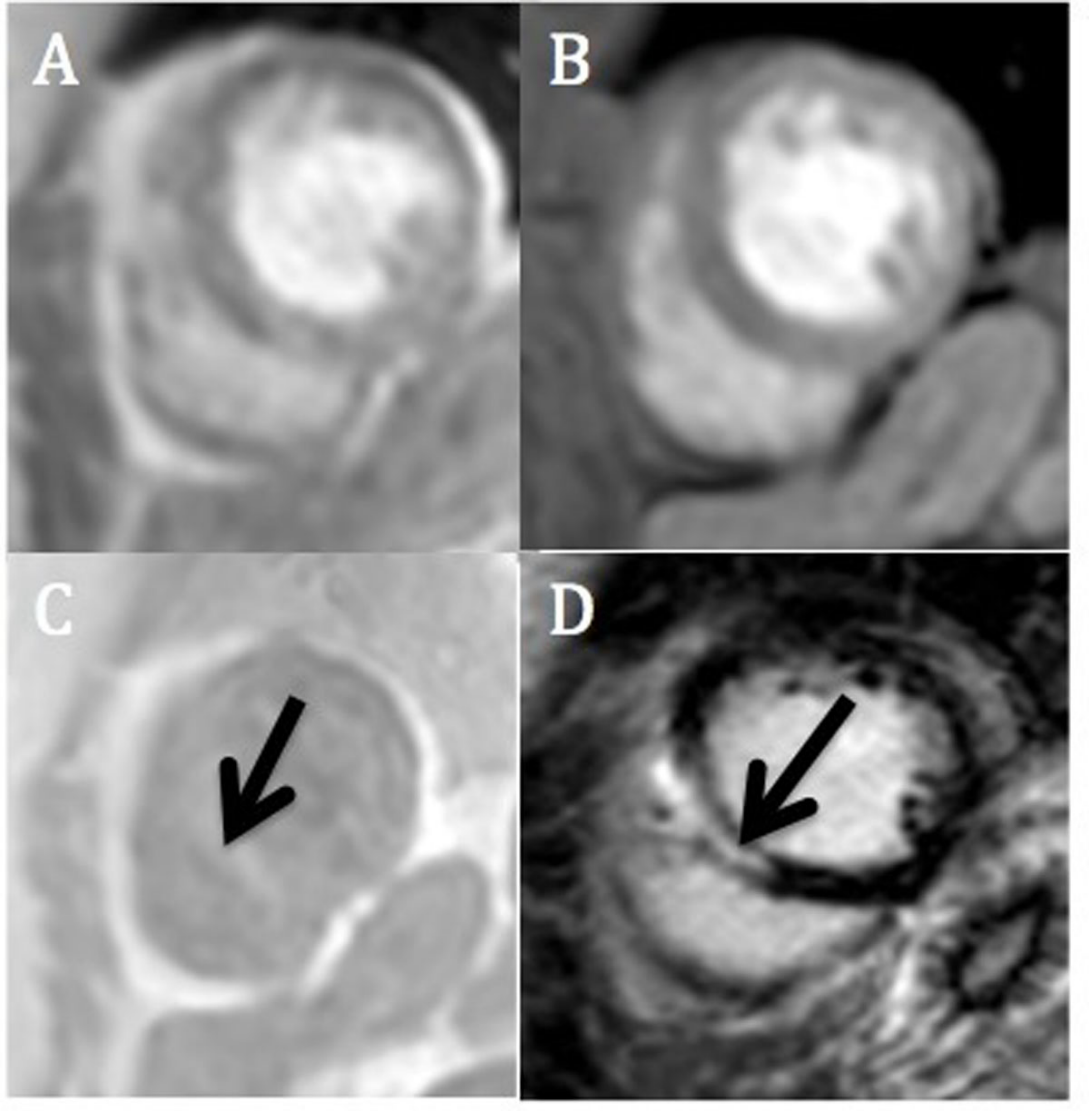
A) UTE with TE of 0.6 ms; B) UTE with TE of 6 ms; C) Subtracted image (black arrow); D) LGE image with myocardial scar (black arrow).

